# Natural Products Targeting Key Molecular Hallmarks in Gastric Cancer: Focus on Apoptosis, Inflammation, and Chemoresistance

**DOI:** 10.3390/ijms27031347

**Published:** 2026-01-29

**Authors:** Daniel Simancas-Racines, Jaen Cagua-Ordoñez, Jaime Angamarca-Iguago, Juan Marcos Parise-Vasco, Claudia Reytor-González

**Affiliations:** 1 Facultad de Ciencias de la Salud y Bienestar Humano, Universidad Tecnológica Indoamérica, Ambato 180150, Ecuador; 2 Facultad de Salud y Bienestar, Pontificia Universidad Católica del Ecuador, Quito 170143, Ecuador; 3 Facultad de Ciencias de la Salud y Bienestar Humano, Universidad Tecnológica Indoamérica, Quito 170103, Ecuador; 4 Escuela de Medicina, Pontificia Universidad Católica del Ecuador, Santo Domingo 230203, Ecuador

**Keywords:** gastric cancer, natural products, apoptosis, inflammation, chemoresistance, tumor microenvironment, ABC transporters, phytochemicals

## Abstract

Natural products have emerged as promising multi-target agents for addressing the complex biology of gastric cancer, a malignancy characterized by marked molecular heterogeneity, late clinical presentation, and frequent resistance to systemic therapies. This narrative synthesis integrates primarily preclinical evidence, with emerging clinical data, on how naturally derived compounds modulate three central molecular processes that drive gastric tumor progression and therapeutic failure: evasion of programmed cell death, persistent tumor-promoting inflammation, and chemoresistance. Compounds such as curcumin, resveratrol, berberine, ginsenosides, quercetin, and epigallocatechin gallate restore apoptotic competence by shifting the balance between pro-survival and pro-death proteins, destabilizing mitochondrial membranes, promoting cytochrome c release, and activating caspase-dependent pathways. These agents also exert potent anti-inflammatory effects by inhibiting nuclear factor kappa B and signal transducer and activator of transcription signaling, suppressing pro-inflammatory cytokine production, reducing cyclooxygenase activity, and modulating the tumor microenvironment through changes in immune cell behavior. In parallel, multiple natural compounds function as chemo-sensitizers by inhibiting drug efflux transporters, reversing epithelial–mesenchymal transition, attenuating cancer stem cell-associated traits, and suppressing pro-survival signaling pathways that sustain resistance. Collectively, these mechanistic actions highlight the capacity of natural products to simultaneously target interconnected hallmarks of gastric cancer biology. Ongoing advances in formulation strategies may help overcome pharmacokinetic limitations; however, rigorous biomarker-guided studies and well-designed clinical trials remain essential to define the translational relevance of these compounds.

## 1. Introduction

Gastric cancer (GC) remains one of the most challenging oncologic diseases worldwide, ranking as the fifth most frequently diagnosed malignancy and the fifth leading cause of cancer-related mortality [1,2]. According to the latest GLOBOCAN 2022 estimates, approximately 968,784 new cases are reported annually, with a markedly elevated burden in East Asian regions such as Mongolia, Japan, and South Korea [3]. Despite advances in early detection and therapeutic innovation, overall 5-year survival remains poor in most regions, typically ranging between 20% and 30%, largely due to late-stage presentation, aggressive tumor behavior, and limited durable treatment options [4].

The molecular heterogeneity of GC has been extensively characterized through large-scale genomic efforts, most notably by The Cancer Genome Atlas (TCGA) and the Asian Cancer Research Group (ACRG) [5,6]. TCGA defines four principal molecular subtypes—Epstein–Barr virus (EBV)-positive (~9%), microsatellite instability (MSI; 21–23%), genomically stable (GS; ~20%), and chromosomal instability (CIN; ~50%)—each associated with distinct genomic alterations, therapeutic vulnerabilities, and clinical outcomes [7]. Concordance analyses further indicate that TCGA GS, EBV, and CIN subtypes broadly correspond to the ACRG MSS/EMT, MSS/TP53+, and MSS/TP53− categories, respectively [8,9].

EBV-positive tumors are characterized by frequent PIK3CA mutations and extensive DNA hypermethylation, often accompanied by overexpression of immune checkpoint ligands PD-L1 and PD-L2 [10], MSI tumors display a high mutational burden and robust immune activation signatures, which have been associated with favorable responses to immune checkpoint inhibition [7]. In contrast, GS tumors are enriched for CDH1 and RHOA mutations and commonly exhibit diffuse-type histology, while CIN tumors are marked by extensive copy-number alterations, TP53 mutations, and amplification of receptor tyrosine kinases that may be amenable to targeted therapeutic strategies [5].

From a clinical standpoint, more than 70% of patients present with advanced or metastatic disease, substantially limiting opportunities for curative treatment [11,12,13]. Therapeutic resistance further compromises outcomes and is driven by a convergence of mechanisms, including overexpression of multidrug efflux transporters, activation of epithelial–mesenchymal transition (EMT) programs, persistence of cancer stem cell populations, and upregulation of DNA damage repair pathways [14,15,16,17,18]. Although standard treatments—such as platinum-based chemotherapy, trastuzumab in HER2-positive disease, and immune checkpoint inhibitors in selected molecular subsets—have improved outcomes in specific contexts, median survival in metastatic GC rarely exceeds 12 months [11,19,20]. Treatment-related toxicity leading to dose reductions or discontinuation further contributes to suboptimal therapeutic efficacy [21,22].

A defining feature of gastric carcinogenesis is the progressive acquisition of molecular hallmarks that promote malignant transformation, invasion, and resistance to therapy [23]. Among these, dysregulation of programmed cell death—driven by TP53 inactivation, overexpression of anti-apoptotic Bcl-2 family proteins, and impaired death receptor signaling—plays a central role in tumor survival and chemoresistance [5,24,25,26,27,28,29]. In parallel, chronic inflammation constitutes a major etiological and promotional factor in GC, mediated by sustained activation of NF-κB, STAT3, and COX-2 signaling pathways, frequently initiated and perpetuated by *Helicobacter pylori* infection [30,31]. In addition, multifactorial chemoresistance emerges through ABC transporter upregulation, metabolic rewiring, cancer stemness, and enhanced DNA repair capacity [18,32,33,34,35,36]. Additional processes, including redox imbalance and aberrant activation of oncogenic signaling cascades such as PI3K/AKT/mTOR and Wnt/β-catenin, further contribute to disease progression and therapeutic failure.

*Helicobacter pylori* infection represents a critical upstream driver of gastric carcinogenesis and provides a mechanistic link between chronic inflammation and malignant transformation. Persistent colonization induces sustained inflammatory signaling, oxidative DNA damage, epigenetic alterations, and progressive mucosal remodeling along the Correa cascade [30,31]. Importantly, several natural products have demonstrated the capacity to modulate *H. pylori*-associated pathogenic mechanisms, either by exerting direct antibacterial effects or by attenuating inflammation-driven signaling pathways such as NF-κB and STAT3. These properties position natural compounds as potential modulators of early carcinogenic events as well as downstream tumor-promoting processes.

Natural products have historically served as a cornerstone of anticancer drug discovery, with nearly half of currently approved oncologic agents derived from or inspired by natural sources [37,38]. Their intrinsic multi-target activity, relative biological selectivity, and capacity to modulate complex signaling networks confer advantages over single-target synthetic agents, particularly in heterogeneous and adaptive malignancies such as GC [39,40]. In this context, naturally derived compounds have demonstrated promising activity across multiple GC-relevant hallmarks, including restoration of apoptotic signaling, suppression of tumor-promoting inflammation, modulation of oncogenic pathways, and reversal of chemoresistance [41,42,43,44]. The structural diversity of these compounds—shaped by evolutionary selection—enables interactions with molecular targets often inaccessible to conventional medicinal chemistry [45,46,47], while their preferential toxicity toward malignant cells reflects exploitation of cancer-specific metabolic and signaling vulnerabilities [48,49,50,51].

This review provides a comprehensive evaluation of natural products relevant to GC therapeutics, focusing on their ability to modulate three core molecular hallmarks—apoptosis, inflammation, and chemoresistance. It integrates mechanistic evidence from preclinical and clinical studies, examines next-generation delivery systems that enhance bioavailability and tumor targeting, and highlights translational barriers to clinical application. Finally, it outlines future directions including precision medicine strategies and rational combination therapy approaches to accelerate the integration of natural products into evidence-based gastric cancer treatment.

## 2. The Molecular Underpinnings of Gastric Carcinogenesis

Gastric carcinogenesis is a multi-step, multi-factorial process driven by the progressive accumulation of genetic and epigenetic alterations that dysregulate essential cellular programs. These alterations enable the acquisition of core malignant capabilities described as the hallmarks of cancer [52]. In GC, three hallmarks are particularly central to disease progression and therapeutic failure—apoptosis evasion, chronic inflammation, and chemoresistance—each orchestrated by tightly interconnected oncogenic signaling networks. Understanding these molecular processes is essential to elucidating how natural products can modulate them and restore cellular vulnerability to treatment [53]. Table 1 summarizes key molecular targets involved in these hallmarks, highlighting their functional significance and relevance for therapeutic modulation.

### 2.1. Evasion of Apoptosis: Bcl-2 Family Imbalance and Caspase Disruption

Apoptosis is a critical safeguard against malignant transformation, eliminating cells harboring oncogenic mutations or excessive proliferative stress [61]. In GC, the intrinsic (mitochondrial) apoptotic pathway is particularly relevant, as it integrates damage signals triggered by chemotherapy and cellular stress [62]. The Bcl-2 protein family acts as the central rheostat of this pathway: anti-apoptotic members (Bcl-2, Bcl-xL, Mcl-1) preserve mitochondrial integrity, while pro-apoptotic proteins (Bax, Bak, and BH3-only proteins such as Bid and Bim) promote mitochondrial permeabilization [61].

GC frequently exhibits overexpression of anti-apoptotic Bcl-2 proteins (up to 67% of tumors), which elevates the apoptotic threshold and contributes directly to chemoresistance [63,64]. Conversely, downregulation or functional impairment of Bax and Bak prevents mitochondrial outer membrane permeabilization (MOMP), a pivotal step that allows cytochrome c release and apoptosome assembly. Once this process is blocked, caspase-9 and downstream executioner caspases (caspase-3/7) remain inactive, effectively silencing programmed cell death [61].

MOMP is the point of no return in the apoptotic cascade. It allows for the release of intermembrane space proteins, most notably cytochrome c, into the cytosol. Cytosolic cytochrome c then binds to the apoptotic protease-activating factor 1 (Apaf-1), triggering the assembly of a large multiprotein complex called the apoptosome. This complex serves as an activation platform for the initiator caspase, caspase-9. Activated caspase-9 then initiates a proteolytic cascade, cleaving and activating the downstream executioner caspases, primarily caspase-3 and caspase-7. These executioner caspases are the final effectors of apoptosis, responsible for cleaving a multitude of cellular substrates, leading to the characteristic morphological and biochemical features of cell death [61].

This distorted Bax/Bcl-2 balance represents a highly actionable vulnerability, as its correction re-sensitizes cancer cells to intrinsic apoptosis. Importantly, a considerable number of natural compounds converge mechanistically on this pathway by increasing Bax expression and/or suppressing Bcl-2, thereby shifting the rheostat toward a pro-apoptotic state—a recurring and central theme in the natural product literature [65].

### 2.2. The Inflammatory Milieu: NF-κB, Cytokine Networks, and Helicobacter pylori

Chronic inflammation is a defining etiological driver of gastric carcinogenesis and a unifying mechanism linking environmental exposure to tumor initiation and progression [66]. *Helicobacter pylori* infection—implicated in most non-cardia GC cases—initiates the Correa cascade and establishes a persistent inflammatory environment that promotes genomic instability, angiogenesis, and immune evasion [67].

NF-κB serves as the master transcriptional hub coordinating this response. Activation of upstream IκB kinase (IKK) pathways—via *H. pylori*, TNF-α, or IL-1β—leads to nuclear translocation of NF-κB and transcription of hundreds of pro-tumorigenic genes [31]. Key NF-κB targets include Cytokines, IL-6, IL-8, TNF-α, which reinforce inflammation through positive feedback loops [66]. Inflammatory enzymes, COX-2, which enhances prostaglandin production, promotes angiogenesis, and suppresses apoptosis [66]. Anti-apoptotic proteins, Bcl-2 family members, directly linking inflammation to cell survival [31].

This NF-κB-driven program creates a self-sustaining inflammatory loop that fuels malignant progression [68]. Because NF-κB is upstream of COX-2, cytokine expression, and anti-apoptotic signaling, targeting this axis has the potential to simultaneously modulate several hallmarks [69]. This provides a strong mechanistic justification for the anti-inflammatory natural products explored later in the review.

### 2.3. The Challenge of Chemoresistance: ABC Transporters, Hypoxia, and EMT

Chemoresistance is a major barrier to successful GC treatment and arises from an intricate network of adaptive mechanisms [70]. Among these, ATP-binding cassette (ABC) transporters are the best characterized. P-glycoprotein (P-gp/MDR1) and MRP1 actively efflux chemotherapeutic agents, lowering intracellular drug concentrations below cytotoxic thresholds. Their expression is frequently elevated in resistant GC cells and is further inducible by drug exposure, establishing a vicious cycle of acquired resistance [71]. The PI3K/AKT pathway is a key transcriptional regulator of MDR1, linking survival signaling with drug efflux capacity [24].

The tumor microenvironment adds an additional layer of complexity. Hypoxia—resulting from rapid tumor expansion and poor vascularization—stabilizes hypoxia-inducible factor 1α (HIF-1α), which drives the transcription of genes promoting angiogenesis, metabolic reprogramming, cell survival, and drug resistance [70]. HIF-1α directly increases P-gp expression, creating a hypoxia–HIF-1α–ABC transporter axis that reinforces chemotherapy resistance [24].

Epithelial–mesenchymal transition (EMT) further exacerbates resistance. During EMT, epithelial markers are lost, and mesenchymal traits emerge, conferring motility, invasiveness, and a stem-like phenotype [24]. EMT cells exhibit heightened survival signaling, increased efflux pump expression, and diminished susceptibility to apoptosis.

These mechanisms form an interconnected resistance network in which hypoxia induces HIF-1α, HIF-1α enhances EMT and ABC transporter expression, and EMT promotes further chemoresistance. Effective therapeutic approaches must therefore target multiple nodes within this system—precisely the type of multi-target activity that many natural products demonstrate.

### 2.4. Intersecting Oncogenic Drivers: PI3K/AKT/mTOR and Wnt/β-Catenin Signaling

Two oncogenic signaling hubs—PI3K/AKT/mTOR and Wnt/β-catenin—play central roles in orchestrating GC progression and ensuring the persistence of multiple hallmarks. Activation of the PI3K/AKT/mTOR axis, commonly due to PIK3CA mutations or PTEN loss [72,73], enhances proliferation, suppresses apoptosis (e.g., via Bad phosphorylation), and upregulates MDR1 expression, directly promoting chemoresistance [24].

Meanwhile, aberrant Wnt/β-catenin signaling—present in up to 50% of GC cases—stabilizes β-catenin, enabling transcription of proliferative and stemness-associated genes such as *c-MYC* and *CYCLIN D1* [74,75]. This pathway is a major driver of EMT and cancer stem cell maintenance, both strongly tied to metastatic potential and treatment failure.

Importantly, these pathways exhibit substantial crosstalk: AKT inhibits GSK-3β, thereby stabilizing β-catenin and amplifying Wnt signaling [76]. Modulating either hub can therefore disrupt multiple malignant phenotypes simultaneously. This mechanistic architecture provides a strong rationale for natural compounds capable of modulating PI3K/AKT or Wnt/β-catenin, many of which are examined in subsequent sections.

## 3. Natural Products as Modulators of Apoptosis in Gastric Cancer

**Figure 1:**
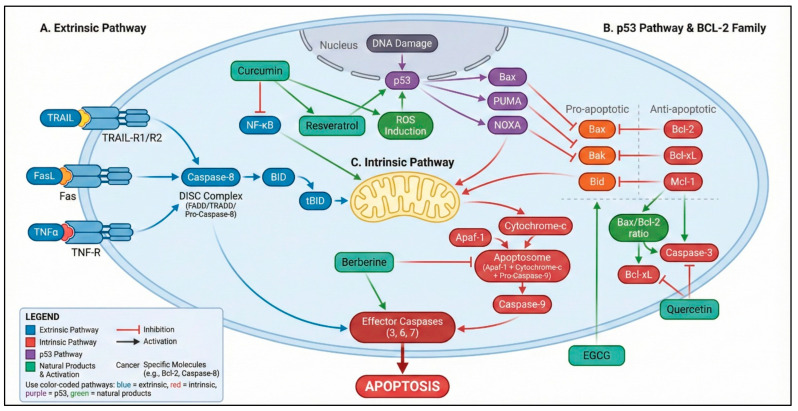
Integrated apoptotic pathways modulated by natural products in gastric cancer. The diagram illustrates the extrinsic apoptotic pathway (TRAIL–TRAIL-R1/R2, Fas–FasL, and TNF-α–TNFR signaling leading to DISC formation and caspase-8 activation) and the intrinsic mitochondrial pathway involving Bax/Bak activation, mitochondrial outer membrane permeabilization, cytochrome c release, apoptosome assembly, and caspase-9 activation. Effector caspases (caspase-3, -6, and -7) execute the apoptotic program. Natural products—including curcumin, resveratrol, quercetin, berberine, and epigallocatechin gallate (EGCG)—modulate key regulatory nodes of these pathways by altering the Bax/Bcl-2 ratio, inhibiting NF-κB signaling, inducing reactive oxygen species (ROS), activating p53-dependent apoptotic programs, and promoting mitochondrial destabilization [88,89,90]. Color coding: blue elements represent the extrinsic pathway, red elements indicate the intrinsic mitochondrial pathway, purple denotes p53-dependent signaling, green boxes represent natural products and their biological effects, and yellow denotes the mitochondrion. Solid arrows indicate activation, whereas T-bar connectors denote inhibitory interactions. Color variations at ligand–receptor binding sites (TRAIL–TRAIL-R1/R2, Fas–FasL, and TNF-α–TNFR) are used for illustrative purposes only and do not indicate distinct biological or signaling differences. Variations in color intensity are applied exclusively for visual clarity and do not imply different biological meanings. Created in BioRender. Reytor, C. (2026) https://BioRender.com/5qt1g8o.

The evasion of apoptosis represents a central survival strategy in gastric cancer (GC) and a major barrier to effective treatment. Natural products offer a diverse array of bioactive molecules capable of reactivating this suppressed cell death machinery through coordinated modulation of mitochondrial integrity, Bcl-2 family dynamics, caspase activation, and upstream tumor suppressor pathways (Figure 1) [23,77]. Collectively, these compounds act on key apoptotic checkpoints that are recurrently dysregulated in GC, complementing the molecular vulnerabilities described in the previous section (Table 2). Curcumin, resveratrol, berberine, quercetin, EGCG, and ginsenoside Rg3 represent the main natural products evaluated in gastric cancer, with their chemical structures summarized in Appendix A.

**Table 2:** Representative Natural Products and Their Apoptosis-Modulating Mechanisms in Gastric Cancer.

Natural Product	Chemical Class	Hallmarks Targeted	Key Mechanistic Actions	Key References
Curcumin	Polyphenol	Apoptosis, Inflammation, Chemoresistance	Bax/Bcl-2 modulation; MOMP induction; caspase-9/3 activation; NF-κB inhibition; MDR1 suppression	Yang et al. (2024) [78]; W Liu (2024) [79]; Ren et al. (2025) [80]
Resveratrol	Stilbenoid	Apoptosis, Inflammation, Chemoresistance	p53 activation; Bax/Bcl-2 modulation; PI3K/AKT inhibition; NF-κB suppression	Warias et al. (2024) [81]
Betulinic Acid	Triterpenoid	Apoptosis	Mitochondrial depolarization; cytochrome-c release; caspase-9/3 activation	Chen et al. (2020) [82]
Ginsenosides	Triterpenoid Saponins	Apoptosis, Chemoresistance	MMP loss; Bax translocation; cytochrome-c release; caspases activation; EMT attenuation	Cui (2025) [83]
Oridonin	Diterpenoid	Apoptosis, Cell Cycle Arrest	Bax/Bcl-2 modulation; cytochrome-c release; caspase activation; G0/G1 or G2/M arrest	Fakhri et al. (2022) [84]
Berberine	Alkaloid	Apoptosis, Inflammation, Chemoresistance	NF-κB inhibition; caspase-3/8/9 activation; MDR1 suppression	Xu et al. (2022) [85]; Kou et al. (2020) [86]
Quercetin	Flavonoid	Apoptosis, Inflammation, Chemoresistance	Mitochondrial dysfunction; JNK/p38 activation; COX-2 reduction; HIF-1α inhibition	Xie et al. (2025) [87]
EGCG	Flavonoid	Apoptosis, Inflammation	STAT3 inhibition; NF-κB suppression; decreased VEGF; mitochondrial ROS induction	Cui (2025) [83]
Celastrol	Triterpenoid	Apoptosis, Inflammation	ROS–JNK activation; cytokine suppression (TNF-α, IL-8)	Fakhri et al. (2022) [84]

### 3.1. Curcumin: Targeting Mitochondrial Vulnerabilities

Curcumin, the main curcuminoid from Curcuma longa, is one of the most extensively studied apoptosis-modulating natural compounds. In GC cell lines, curcumin reduces viability in a dose-dependent manner and induces significant apoptosis through coordinated mitochondrial disruption [91,92]. Curcumin shifts the Bax/Bcl-2 equilibrium toward a pro-apoptotic state, triggers mitochondrial membrane depolarization, and promotes cytochrome-c release, ultimately activating caspase-9 and caspase-3 [93,94]. This integrated mitochondrial and caspase activation has also been observed in vivo, where curcumin treatment suppresses xenograft tumor growth and increases intratumoral caspase-3 expression [95]. Curcumin’s ability to simultaneously inhibit NF-κB and MDR1 further strengthens its therapeutic relevance by lowering inflammatory and chemoresistant thresholds [96].

### 3.2. Resveratrol: Dual Engagement of p53 and Mitochondrial Pathways

Resveratrol demonstrates a multi-targeted pro-apoptotic effect, acting on both the mitochondrial pathway and upstream tumor suppressor mechanisms. In GC models, resveratrol increases Bax and decreases Bcl-2 expression, promotes mitochondrial permeabilization, and activates the caspase cascade [97]. Importantly, resveratrol enhances p53 activation, reinforcing transcriptional upregulation of pro-apoptotic genes and strengthening mitochondrial commitment to apoptosis [98]. Suppression of PI3K/AKT and NF-κB signaling provides a complementary survival blockade, creating a convergent pro-death signal that lowers the apoptotic threshold [99]. Although in vivo GC-specific studies remain limited, the mechanistic rationale and cross-cancer validation support its translational potential.

### 3.3. Betulinic Acid and Ginsenosides: Direct Mitochondrial Initiators

Betulinic acid (BA) exerts selective cytotoxicity against cancer cells by directly impairing mitochondrial stability. BA induces rapid loss of mitochondrial membrane potential, promotes cytochrome-c release, and triggers activation of caspase-9 and caspase-3 [100,101,102]. BA also shifts the Bax/Bcl-2 ratio toward apoptosis, a mechanism confirmed in GC cell models and xenografts [103].

Ginsenosides—including Rg3, Rh1, Rk1, and M1—activate mitochondrial apoptosis by promoting Bax translocation, reducing MMP, and triggering caspase-dependent cell death [85,86]. Ginsenosides also attenuate EMT and reverse chemoresistant phenotypes, giving them a dual apoptotic and anti-resistance profile validated across multiple in vivo GC models [104].

### 3.4. Oridonin: Threshold-Dependent Cell Death Induction

Oridonin displays a concentration-dependent dichotomy of biological responses. At lower doses, it induces cell cycle arrest and senescence, whereas higher concentrations activate the intrinsic apoptotic cascade through Bax upregulation, Bcl-2 suppression, cytochrome-c release, and caspase-9/3 activation [105,106]. These effects have been corroborated in several GC xenograft models [107]. Oridonin’s clear dose–response transition between cytostasis and apoptosis provides a rational basis for therapeutic dose optimization.

## 4. Countering the Inflammatory Tumor Microenvironment with Natural Compounds

**Figure 2:**
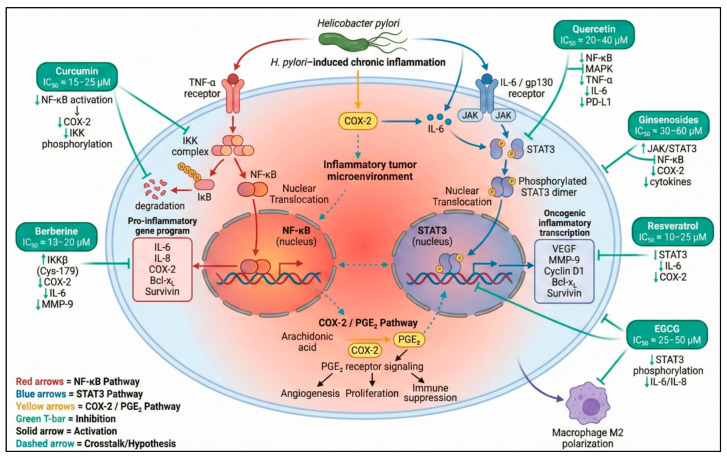
Inflammatory pathways and anti-inflammatory mechanisms modulated by natural products in gastric cancer. This schematic illustrates the central inflammatory signaling axes involved in gastric carcinogenesis, including NF-κB activation through TNF-α/IKK signaling, COX-2-derived prostaglandin pathways, and IL-6-mediated JAK/STAT3 oncogenic transcription. Key natural compounds—curcumin, berberine, quercetin, EGCG, resveratrol, and ginsenosides—exert inhibitory effects on these nodes by reducing IKK phosphorylation, blocking STAT3 activation, suppressing COX-2 expression, decreasing cytokine release (TNF-α, IL-6, IL-1β), and modulating immune cell polarization. Crosstalk among NF-κB, COX-2/PGE_2_, and STAT3 pathways is shown to highlight the interconnected inflammatory microenvironment characteristic of gastric cancer [31,32,110,111,112,113]. Symbols and color coding: Molecular icons represent receptors, signaling complexes, transcription factors, and nuclear translocation for illustrative purposes. Colored arrows indicate pathway activation or inhibition as depicted in the figure. Green upward and downward arrows (↑/↓) denote upregulation or downregulation of the indicated molecular targets, respectively. Created in BioRender. Reytor, C. (2026) https://BioRender.com/saenqa1.

Chronic inflammation is a defining feature of gastric carcinogenesis and a major driver of tumor initiation, progression, and immune evasion [67]. Persistent activation of inflammatory signaling pathways such as NF-κB and STAT3 establishes a self-reinforcing tumor microenvironment (TME) characterized by sustained cytokine production, macrophage reprogramming toward immunosuppressive phenotypes, and attenuation of cytotoxic immune surveillance [68]. Natural products exert potent anti-inflammatory and immunomodulatory effects that disrupt this tumor-promoting inflammatory loop by inhibiting key signaling hubs, reshaping cytokine networks, and actively reprogramming the immune composition of the gastric TME [108]. Through these coordinated mechanisms, natural compounds suppress inflammation-driven malignant transformation while restoring antitumor immune competence, thereby creating a biological context more permissive to immune-mediated tumor control [109] (Figure 2). A detailed summary of preclinical studies supporting the anti-inflammatory and immunomodulatory effects of these natural products in gastric cancer models is provided in Appendix B.

### 4.1. Curcumin and Berberine: Targeting the NF-κB-Driven Inflammatory and Immunosuppressive Axis

Curcumin and berberine act as archetypal inhibitors of the NF-κB pathway, a central orchestrator of inflammation, survival signaling, immune evasion, and macrophage polarization in gastric cancer. Inhibition of this master regulator yields dual therapeutic benefits by simultaneously attenuating inflammatory signaling and lowering the apoptotic threshold of tumor cells, while also mitigating NF-κB-dependent immune suppression [114,115].

Curcumin exerts robust anti-inflammatory activity largely through suppression of NF-κB activation [116,117]. n preclinical *Helicobacter pylori*-induced gastritis models, curcumin significantly reduces mucosal inflammation and pro-tumorigenic cytokine production, interrupting early steps of the Correa cascade [116]. By diminishing NF-κB-regulated expression of COX-2, IL-6, and TNF-α, curcumin weakens the inflammatory milieu that supports gastric tumor initiation, progression, and immune escape [118,119]. Emerging evidence further suggests that curcumin-mediated NF-κB inhibition indirectly limits the expansion of immunosuppressive myeloid populations, thereby favoring a shift toward a more immune-permissive TME [120,121].

Berberine complements this mechanism through a highly specific mode of NF-κB inhibition. By covalently modifying Cys-179 of the IKKβ subunit, berberine prevents IκBα phosphorylation and degradation, maintaining NF-κB in its inactive cytosolic state [122]. The downstream impact includes suppression of COX-2, MMP-9, cyclin D1, survivin, and Bcl-xL—genes that collectively sustain inflammatory signaling, proliferation, resistance to apoptosis, and immune evasion in gastric cancer [122,123]. Through this broad repression of NF-κB-dependent programs, berberine disrupts the inflammation–survival–immunosuppression axis that underlies GC progression and therapeutic resistance [124,125].

### 4.2. Flavonoids (Quercetin, EGCG): Immunomodulatory Reprogramming of the Tumor Microenvironment

Flavonoids such as quercetin and epigallocatechin-3-gallate (EGCG) extend their therapeutic effects beyond direct anti-inflammatory activity by actively reprogramming the immune landscape of the gastric TME. Rather than acting solely on tumor cells, these compounds modulate macrophage polarization, suppress immunosuppressive cytokines, and enhance cytotoxic immune cell function, thereby reshaping both innate and adaptive immune responses [126,127].

Quercetin inhibits NF-κB and MAPK signaling, leading to reduced expression of TNF-α, IL-6, and COX-2, while simultaneously promoting a shift from M2-like tumor-associated macrophages (TAMs) toward an antitumor M1 phenotype [128,129]. This macrophage re-education is accompanied by enhanced CD8^+^ T-cell activity and reduced regulatory T-cell abundance, indicating a broader restoration of antitumor immune surveillance. Importantly, quercetin also suppresses PD-L1 expression on tumor cells, a mechanism with direct relevance to immune checkpoint blockade and the potential to sensitize gastric tumors to PD-1/PD-L1-targeted therapies [130,131].

EGCG exhibits similarly extensive immunomodulatory effects. In gastric cancer models, EGCG inhibits IL-6/STAT3 signaling, resulting in decreased expression of downstream angiogenic and immunosuppressive mediators such as VEGF [112,132]. EGCG further limits macrophage infiltration by downregulating CCL2 and CSF-1 and prevents M2 polarization through miR-16-mediated suppression of NF-κB signaling within macrophages [126]. Through these coordinated actions, EGCG dismantles the immunosuppressive TME that promotes tumor survival and therapeutic resistance, aligning with the growing interest in flavonoids as immunological co-adjuvants capable of enhancing responses to immune checkpoint inhibitors [126].

### 4.3. Celastrol: Disrupting Cytokine Networks and the Inflammatory–Immune Interface

Celastrol, a triterpenoid derived from *Tripterygium wilfordii*, displays potent anti-inflammatory activity in gastric cancer through disruption of cytokine networks and inhibition of tumor-supportive inflammatory mediators [133]. In GC cell models, celastrol markedly suppresses TNF-α and IL-8 secretion while downregulating Biglycan (BGN), a proteoglycan that amplifies innate immune signaling and cytokine release [134]. Experimental overexpression of BGN rescues cytokine production, confirming its role as a functional target [135].

Although necroptosis has been implicated as part of celastrol’s cytotoxic profile, its anti-inflammatory effect in GC is most consistently associated with downregulation of BGN and inhibition of inflammatory cytokine release more consistently linked to BGN/cytokines in GC models [134]. By simultaneously inducing tumor cell death and attenuating cytokine-driven inflammation, celastrol disrupts a key interface between cellular and microenvironmental processes that sustain gastric carcinogenesis [136,137].

Collectively, these findings position anti-inflammatory and immunomodulatory natural products not as standalone immunotherapies, but as biologically rational co-adjuvants capable of reshaping the gastric tumor microenvironment to improve responsiveness to immune checkpoint inhibition.

## 5. Overcoming Chemoresistance: The Role of Natural Products as Chemo-Sensitizers

**Figure 3:**
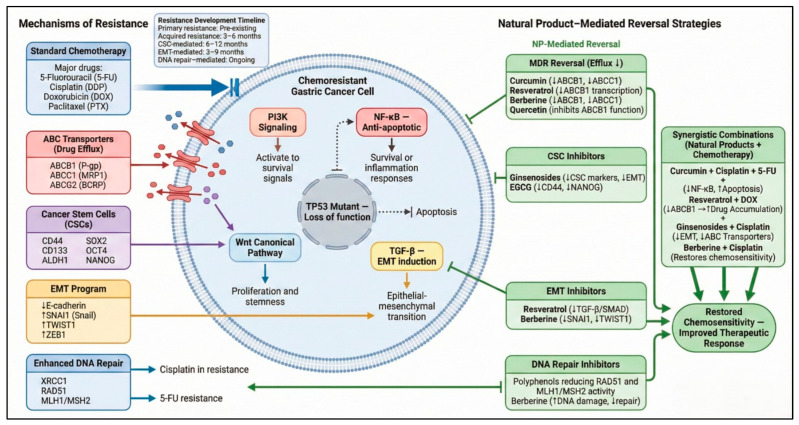
Chemoresistance Mechanisms and Natural Product Reversal Strategies in Gastric Cancer. This figure illustrates the major molecular pathways driving chemoresistance in gastric cancer—including drug efflux via ABC transporters (P-gp/ABCB1, MRP1/ABCC1, ABCG2), cancer stem cell-associated persistence (CD44, CD133, ALDH1), EMT activation (Snail, Twist, Zeb1), enhanced DNA repair (RAD51, MLH1/MSH2), and pro-survival signaling through PI3K/AKT, NF-κB, TGF-β/Smad, and Wnt/β-catenin. Natural products counteract these mechanisms by inhibiting drug efflux (curcumin, resveratrol, berberine, quercetin), suppressing CSC and EMT programs (ginsenosides, EGCG), attenuating pro-survival pathways, and disrupting DNA repair networks. Synergistic combinations with cisplatin, 5-FU, doxorubicin, or oxaliplatin restore chemosensitivity and enhance therapeutic response. Solid arrows indicate activation, dotted arrows reduction or crosstalk, and T-bars denote inhibition [140,141,142,143,144,145]. Arrows within text boxes indicate reported increases or decreases in molecular activity or expression (↑/↓). Created in BioRender. Reytor, C. (2026) https://BioRender.com/5a62wlg.

Chemoresistance remains one of the principal barriers to successful treatment of advanced gastric cancer, contributing directly to poor survival outcomes and high recurrence rates [18]. Natural products are emerging as promising chemo-sensitizers capable of overcoming resistance through multi-target actions that simultaneously intercept the cellular and microenvironmental mechanisms that sustain the resistant phenotype [138]. Their pleiotropic nature allows them to interfere with efflux pumps, restore apoptosis, suppress survival signaling, and target epithelial–mesenchymal transition (EMT) and cancer stem cell (CSC) traits—features that make resistance so difficult to reverse with single-target synthetic agents [139] (Figure 3). Preclinical studies demonstrating the ability of natural products to reverse chemoresistance and enhance the efficacy of conventional chemotherapy in gastric cancer models are summarized in Appendix C.

### 5.1. Mechanisms of Action: Disrupting the Architecture of Chemoresistance

The multifactorial design of natural products is a strategic advantage in combatting chemoresistance. Rather than inhibiting a single node, these compounds mount a multipronged attack that destabilizes the resistant phenotype at its foundations [54].

- Inhibition of ABC Transporters: Overexpression of ATP-binding cassette (ABC) efflux pumps—particularly P-glycoprotein (P-gp/MDR1) and MRP1—is a dominant mechanism of multidrug resistance. Flavonoids, alkaloids, and terpenoids have been shown to (i) Directly inhibit ATPase activity of P-gp/MDR1, (ii) block drug efflux, (iii) downregulate MDR1/MRP1 transcription, leading to increased intracellular drug accumulation and restoration of cytotoxicity [146].
- Restoration of Apoptotic Sensitivity: Chemoresistant cells typically display heightened thresholds for apoptosis due to elevated Bcl-2 expression or impaired mitochondrial signaling. Many natural products reverse this state by (i) Shifting the Bax/Bcl-2 ratio, (ii) promoting cytochrome-c release, (iii) activating caspase-3/8/9, thereby lowering the apoptotic threshold and enabling chemotherapeutic agents to trigger cell death more effectively [70].
- Targeting EMT and Cancer Stem Cells: EMT contributes to invasiveness, survival, and drug resistance, while CSCs act as reservoirs for tumor regrowth. Natural compounds have demonstrated the ability to: (i) Reverse EMT by upregulating E-cadherin and suppressing Snail/Twist/ZEB1, (ii) reduce CSC markers such as CD44, ALDH1, and CD133, (iii) impair sphere formation and self-renewal capacity [147]. Berberine, in particular, has shown capacity to reduce CSC-like populations in various cancer models [54].
- Suppression of Pro-survival Signaling: Hyperactivation of pathways such as PI3K/AKT/mTOR provides survival cues that blunt chemotherapy-induced damage. Natural products including berberine and resveratrol inhibit these pathways, dismantling the signaling support that sustains resistance [86].

This broad-spectrum mechanistic action makes natural products fundamentally advantageous: while a tumor cell may compensate for inhibition of one pathway, it is far more difficult to counteract simultaneous suppression of drug efflux, survival signaling, EMT, and apoptotic evasion.

### 5.2. Synergistic Interactions: Enhancing Conventional Chemotherapy

Compelling preclinical evidence supports the synergistic integration of natural products with frontline chemotherapeutic agents such as cisplatin (DDP), 5-fluorouracil (5-FU), and oxaliplatin.

- Curcumin reverses 5-FU resistance by inhibiting NF-κB, thereby restoring apoptosis and significantly enhancing cytotoxicity. When combined with the FP regimen (5-FU + cisplatin), curcumin displays potent synergy in MGC-803 cells—most pronounced at lower chemotherapy doses—suggesting its utility in dose reduction strategies. This synergy is mediated through (i) Caspase-3/8 activation, (ii) downregulation of Bcl-2, (iii) mitochondrial depolarization [148].
- In AGS cells, resveratrol enhances doxorubicin sensitivity by downregulating MDR1 and MRP1 expression, effectively targeting the efflux-driven mechanism of resistance [149]. This is consistent with its broader capacity to modulate PI3K/AKT and NF-κB pathways.
- Ginsenosides demonstrate consistent synergy with platinum agents: Ginsenoside Rk1 significantly enhances cisplatin and oxaliplatin efficacy in vivo by inhibiting tumor growth [150]. Ginsenoside Rg3, in combination with a STING agonist, reverses cisplatin resistance by suppressing EMT and downregulating resistance-associated proteins [151]. These findings support the inclusion of ginsenosides in combination regimens aimed at reversing EMT-driven chemoresistance.
- Berberine exhibits some of the strongest chemo-sensitizing effects among natural compounds. In cisplatin-resistant GC cells, berberine restores sensitivity by (i) Downregulating MDR1/MRP1, (ii) inducing mitochondrial apoptosis, (iii) inhibiting PI3K/AKT/mTOR signaling [86]. These effects have been validated both in vitro and in vivo, positioning berberine as one of the most clinically promising natural sensitizers.

## 6. Translational Barriers and Future Directions

Despite compelling mechanistic evidence supporting the antitumor activity of natural products in gastric cancer, their translation into clinically validated therapies remains limited. The gap between robust in vitro efficacy and modest in vivo or clinical outcomes reflects a constellation of scientific, pharmacological, regulatory, and economic barriers that continue to constrain progress. Addressing these limitations is essential to contextualize the mechanistic findings discussed throughout this review and to guide future development strategies.

### 6.1. Persistent Barriers to Clinical Translation

One of the most significant challenges is the lack of chemical standardization and batch-to-batch consistency in natural product preparations. Variability arising from differences in plant species or subspecies, geographic origin, cultivation conditions, harvesting, storage, and extraction protocols results in substantial heterogeneity in bioactive compound content [152]. Without rigorously standardized formulations manufactured under Good Manufacturing Practice (GMP) conditions, it remains difficult to attribute biological or clinical effects to a defined intervention, contributing to inconsistent and non-reproducible clinical findings [153].

Pharmacokinetic limitations represent an additional and critical obstacle. Many phytochemicals exhibit poor aqueous solubility, rapid metabolism, and limited tissue penetration, leading to a pronounced disconnect between potent in vitro IC_50_ values and insufficient in vivo exposure at tumor sites. As a consequence, promising compounds may fail to engage their molecular targets at therapeutically relevant concentrations in clinical settings, despite strong mechanistic rationale [153].

Safety considerations further complicate clinical translation. The widespread perception that “natural” equates to “safe” is misleading. At pharmacologically active doses, several natural compounds have been associated with hepatotoxicity, nephrotoxicity, mitochondrial dysfunction, and hematologic alterations [154]. Of particular concern are drug–herb interactions mediated by cytochrome P450 enzymes, especially CYP3A4 and CYP2C9, which metabolize many chemotherapeutic agents. Inhibition or induction of these enzymes by natural products can result in clinically significant alterations in drug exposure, increasing the risk of toxicity or therapeutic failure [155].

Finally, limited financial incentives and challenges related to intellectual property protection have curtailed pharmaceutical investment in natural product development. The lack of patentability of core chemical structures translates into fewer high-quality randomized clinical trials, insufficient pharmacokinetic and pharmacodynamic studies, and delayed adoption of advanced formulation strategies, despite strong biological plausibility [153].

### 6.2. Delivery-Related Challenges: A Brief Perspective

In addition to the barriers outlined above, delivery-related limitations play a contributory role in the restricted clinical performance of many natural compounds. Several agents highlighted in this review—including curcumin, resveratrol, ginsenosides, and epigallocatechin gallate (EGCG)—exhibit particularly low oral bioavailability due to poor solubility, physicochemical instability, and extensive first-pass metabolism [156,157,158]. Curcumin, for example, often remains undetectable in plasma following oral administration despite potent cytotoxic effects in gastric cancer cell lines, reflecting rapid glucuronidation, sulfation, and limited systemic exposure [159,160].

Nanotechnology-based delivery systems have been explored as enabling tools to partially address these pharmacokinetic constraints by improving compound stability, solubility, and circulation time [161]. Importantly, such approaches do not modify the intrinsic molecular mechanisms of action discussed in earlier sections but rather seek to enhance delivery efficiency and tumor accessibility [162]. Nanoformulations can protect labile phytochemicals from premature degradation and promote preferential tumor accumulation through enhanced permeability and retention effects, particularly for particles below 200 nm [163]. Advanced carrier designs may further allow controlled or stimuli-responsive release in response to tumor-associated cues such as acidic pH, redox imbalance, or elevated reactive oxygen species [164].

Preclinical studies have demonstrated improved antitumor efficacy of nano-encapsulated natural products in gastric cancer models, including liposomal curcumin, resveratrol-loaded solid lipid nanoparticles, ginsenoside-containing polymeric micelles, and EGCG-loaded chitosan-based nanoparticles [165,166]. Moreover, the clinical use of nanoformulated chemotherapeutics in gastric cancer, such as liposomal irinotecan (Onivyde^®^, Boulogne-Billancourt, France) and liposomal paclitaxel (Lipusu^®^, Nanjing, China), provides proof-of-concept that nanocarrier-based delivery is feasible within this disease context [167,168]. Nevertheless, these strategies should be viewed as supportive measures to address delivery-related limitations rather than as central therapeutic drivers.

### 6.3. Integrating Natural Products into Precision Oncology Frameworks

The therapeutic landscape of gastric cancer is increasingly shaped by precision oncology, with treatment decisions guided by molecular features such as HER2 amplification, microsatellite instability, PD-L1 expression, Epstein–Barr virus positivity, and alterations in key oncogenic pathways. Within this context, the traditionally perceived lack of specificity of natural products may represent a relative advantage, given their capacity to modulate multiple interconnected signaling networks involved in apoptosis dysregulation, chronic inflammation, and chemoresistance [169,170,171].

Future development efforts should prioritize biomarker-driven strategies to identify patient subsets most likely to benefit from specific natural compounds [172]. Predictive signatures related to inflammatory signaling, survival pathway activation, epithelial–mesenchymal transition, or cancer stem cell traits may guide rational selection and avoid non-specific application [173,174]. In parallel, natural products are more plausibly positioned as components of combination strategies rather than as monotherapies for advanced disease—acting as chemo-sensitizers, immunomodulatory co-adjuvants, or metabolic modulators that complement established cytotoxic, targeted, or immunotherapeutic agents [70].

### 6.4. From Preclinical Promise to Clinical Validation

Despite encouraging preclinical data, the clinical evidence base supporting natural products in gastric cancer remains fragmented and methodologically limited. Progress toward clinical credibility will require development pipelines that adhere to the same scientific and regulatory standards applied to synthetic small molecules [153,175]. This includes rigorous chemical standardization, GMP-compliant manufacturing, comprehensive pharmacokinetic and pharmacodynamic characterization, and well-designed randomized clinical trials incorporating biomarker-guided patient stratification and objective measures of target engagement.

The principal limitation facing the field is therefore not a shortage of biologically active compounds, but an absence of pharmaceutical-grade rigor. Natural products possess genuine potential as multi-target modulators capable of addressing key molecular hallmarks of gastric cancer; however, their successful integration into evidence-based oncology will depend on disciplined translational strategies and high-quality clinical validation [153,176].

## 7. Conclusions

Gastric cancer remains a major global health challenge due to its pronounced molecular heterogeneity, aggressive clinical behavior, and the frequent development of resistance to standard therapies. The convergence of three interrelated molecular hallmarks—evasion of apoptosis, chronic inflammation, and multidimensional chemoresistance—creates a highly adaptive tumor ecosystem that limits the effectiveness of single-target therapeutic strategies. These features underscore the need for interventions capable of modulating interconnected signaling networks that collectively sustain tumor survival, progression, and treatment failure.

The evidence synthesized in this review indicates that natural products represent a mechanistically diverse class of multi-target modulators capable of engaging these hallmarks at multiple levels. Compounds such as curcumin, resveratrol, berberine, ginsenosides, quercetin, and EGCG have been shown in preclinical models to restore apoptotic signaling, suppress pro-tumorigenic inflammatory pathways, and attenuate key mechanisms of chemoresistance, including efflux pump activity, epithelial–mesenchymal transition, and cancer stem cell-associated traits. However, the clinical integration of these agents remains limited, not by a lack of biologically active compounds, but by insufficient pharmaceutical-grade rigor, including challenges related to bioavailability, standardization, pharmacokinetics, and clinical validation. Future progress will depend on disciplined translational strategies, biomarker-guided patient selection, and well-designed clinical trials that position natural products as rational adjuncts—rather than replacements—to established cytotoxic, targeted, or immunotherapeutic regimens. Within such frameworks, natural products may contribute meaningfully to more biologically informed and evidence-based approaches for managing gastric cancer.

## Figures and Tables

**Table 1:** Molecular Targets Implicated in Key Hallmarks of Gastric Cancer and Their Functional Roles.

Molecular Target	Protein Family/Type	Primary Function in Gastric Cancer	Associated Hallmark(s)	Key References
Bcl-2	Anti-apoptotic Bcl-2 family	Sequesters pro-apoptotic proteins; maintains mitochondrial integrity; promotes survival and chemoresistance.	Apoptosis evasion; chemoresistance	Zhou et al. (2025) [54]
Bax	Pro-apoptotic Bcl-2 family	Promotes mitochondrial outer membrane permeabilization (MOMP) and cytochrome c release; often downregulated or inhibited.	Apoptosis evasion	Amjad et al. (2022) [55]
Caspase-9/Caspase-3	Initiator/executioner caspases	Execute apoptotic cascade; their inactivation blocks programmed cell death and enhances resistance to therapy.	Apoptosis evasion	Amjad et al. (2022) [55]
NF-κB	Transcription factor	Induces pro-inflammatory cytokines (IL-6, TNF-α), COX-2, and anti-apoptotic genes; central driver of tumor-promoting inflammation.	Inflammation; apoptosis evasion; chemoresistance	Chaithongyot et al. (2021) [31]
COX-2	Cyclooxygenase enzyme	Produces prostaglandins that promote inflammation, angiogenesis, and inhibit apoptosis; overexpressed in GC.	Inflammation	Xu et al. (2024) [56]
IL-6	Cytokine	Activates STAT3 and promotes proliferation, angiogenesis, and immune evasion.	Inflammation; tumor progression	Yu et al. (2024) [57]
P-gp (MDR1/ABCB1)	ABC transporter	Effluxes chemotherapeutic agents, reducing intracellular drug accumulation; major mediator of multidrug resistance.	Chemoresistance	Li et al. (2024) [14]
HIF-1α	Transcription factor	Mediates hypoxia response; upregulates genes promoting angiogenesis, glycolysis, and MDR1 expression.	Chemoresistance; metastasis	Albano et al. (2025) [58]
PI3K/AKT	Kinase signaling cascade	Central survival pathway; inhibits apoptosis, enhances proliferation, and induces MDR1 expression.	Apoptosis evasion; chemoresistance	Morgos et al. (2024) [59]
β-catenin	Transcriptional co-activator	Drives Wnt-mediated transcription promoting proliferation, EMT, and cancer stemness.	Chemoresistance; apoptosis evasion	Lei et al. (2022) [60]

MOMP: mitochondrial outer membrane permeabilization; ABC transporters: ATP-binding cassette transporters; EMT: epithelial–mesenchymal transition. Molecular targets included were selected based on consistent evidence from peer-reviewed studies demonstrating reproducible involvement in at least one major hallmark of gastric cancer (apoptosis evasion, inflammation, or chemoresistance). Their functions are derived from experimental and translational studies in gastric cancer cell lines, xenograft models, and human tumor analyses.

**Figure A1:**
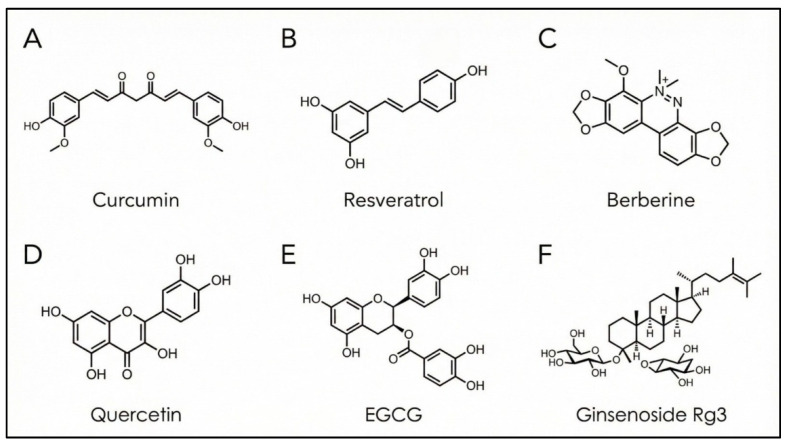
Chemical structures of representative natural products discussed in this review. (**A**) Curcumin; (**B**) Resveratrol; (**C**) Berberine; (**D**) Quercetin; (**E**) Epigallocatechin-3-gallate (EGCG); (**F**) Ginsenoside Rg3. Created in BioRender. Reytor, C. (2026) https://BioRender.com/ojozp53.

## Data Availability

No new data were created or analyzed in this study.

## Appendix A. Chemical Structures of Representative Natural Products

This appendix presents the chemical structures of representative natural products discussed in the main text. These structures are provided for reference and do not imply structure–activity relationships beyond those addressed in the mechanistic sections.

## Appendix B. Preclinical Evidence Supporting the Anticancer Activity of Natural Products in Gastric Cancer

This appendix provides a structured overview of representative preclinical studies supporting the mechanistic effects of natural products discussed in the main text. These data complement the mechanistic sections by summarizing experimental models, dosing conditions, primary biological effects, and molecular pathways involved, without interrupting the narrative flow of the manuscript.

**Table A1:** Preclinical evidence supporting anticancer activity of natural products in gastric cancer.

Natural Product	Experimental Model (Cell Line/Animal)	Dose/Concentration	Main Effects	Molecular Pathways	Reference
Curcumin	Gastric cancer cell lines (in vitro); preclinical animal models	Micromolar concentrations in vitro; various dosing in vivo (see source)	Inhibits proliferation, invasion, migration; induces apoptosis; modulates lncRNA and signaling pathways	PI3K/Akt/mTOR; NF-κB; modulation of lncRNAs	Wang B et al. (2024) [92]
Resveratrol	Gastric cancer cell models (in vitro)	Micromolar range (dose–response reported)	Suppresses proliferation; induces apoptosis; modulates pro-survival pathways	Inhibition of PI3K/AKT and associated pathways	Rojo D et al. (2022) [177]
Betulinic acid	Gastric cancer cells (in vitro); xenograft mouse model (in vivo)	Dose–response concentrations in vitro; in vivo administration schemes reported	Decreases proliferation; induces apoptosis; reduces EMT and metastatic potential	Mitochondrial apoptosis; EMT-associated signaling	Che Y et al. [82]
Ginsenoside Rg3	Cisplatin-resistant gastric cancer cells (SGC-7901/DDP) in vitro + in vivo assays	Dosing varied (e.g., c-di-AMP + RG3 in combination; see study)	Synergistic inhibition of proliferation, migration, invasion, and EMT; reversal of chemoresistance	EMT suppression; stemness reduction; immune pathway modulation	Lu Z et al. [178]
Oridonin	GC cell lines (in vitro)	Micromolar concentrations (effective range reported)	Induces apoptosis; reduces proliferation; alters expression of apoptosis-associated genes	Caspase-dependent apoptosis, transcriptomic effects	Ren D et al. [179]
Berberine	BGC-823 gastric cancer cells (in vitro)	5–40 μM in typical cellular assays	Suppresses proliferation and invasion; impacts survival and inflammatory signaling	NF-κB inhibition; apoptosis modulation	Tian Y et al. [180]
Quercetin	AGS and HGC-27 gastric cancer cell lines (in vitro)	50–200 μM (AGS); 100–300 μM (HGC-27)	Reduces migration and invasion; inhibits EMT; suppresses proliferative signaling	MAPK/ERK; EMT regulation	Deng H et al. [90]
EGCG	Gastric cancer cells (in vitro); mouse tumor models (in vivo)	5–40 μM (in vitro); 1.5 mg/day/mouse (in vivo) in some studies	Inhibits proliferation; increases apoptosis; reduces tumor growth in vivo	Apoptosis regulation; proliferative signaling pathways	Jang J et al. [181]
Celastrol	HGC-27, AGS gastric cancer cell models (in vitro)	0.5 μM for 24 h (effective in cited study)	Suppresses cytokine release (TNF-α/IL-8); anti-inflammatory and cytotoxic effects	Biglycan (BGN) axis; inflammation–cell death interface	Guo D et al. [134]

## Appendix C. Preclinical Evidence Supporting the Anticancer Activity of Natural Products in Gastric Cancer

This appendix summarizes currently registered clinical trials that have evaluated or are evaluating selected natural products in gastric cancer or related precursor conditions, including chronic atrophic gastritis and gastric intestinal metaplasia. These trials provide limited but important clinical context to the preclinical and mechanistic evidence discussed in the main text. The information is presented to acknowledge ongoing translational efforts while recognizing that robust clinical validation remains limited.

**Table A2:** Registered clinical trials involving natural products relevant to gastric cancer.

Natural Product	Trial ID	Phase	Population	Intervention	Outcomes (Primary/Key)	Status
Curcumin	NCT02782949	Phase IIb	Adults with chronic atrophic gastritis and/or gastric intestinal metaplasia (GC prevention setting)	Curcumin vs. placebo (with biomarker/lab assessments; QoL components reported in registry descriptions)	Prevention-oriented endpoints and biomarker/lesion-related assessments (registry-described)	Active, not recruiting
Berberine	NCT07129460	Phase IV	Patients with gastric intestinal metaplasia (risk/precancer context)	Berberine hydrochloride vs. placebo	Efficacy and safety outcomes for gastric intestinal metaplasia (registry-described)	Not yet recruiting
Ginsenoside Rg3	NCT01757366	Phase II	Advanced gastric cancer (treatment setting)	Ginsenoside Rg3 + first-line chemotherapy vs. chemotherapy (as described in registry summaries/aggregators)	Safety and efficacy endpoints (registry-described); no results posted page identified	Unknown/no results posted
Curcumin (supportive care/cachexia context including GC)	NCT05856500	(Not clearly displayed in public snippets; described as prospective controlled study in available documents)	Patients with advanced upper GI tumors including gastric cancer (III–IV) with early cachexia	Creatine + curcumin (oral) on top of basic nutritional support	Inflammation/metabolic and nutrition-related outcomes; QoL/prognosis-related endpoints (per protocol/ICF text)	Not yet recruiting (reported in curated summaries of GC-related curcumin trials)
